# A Step Towards Seascape Scale Conservation: Using Vessel Monitoring Systems (VMS) to Map Fishing Activity

**DOI:** 10.1371/journal.pone.0001111

**Published:** 2007-10-31

**Authors:** Matthew J. Witt, Brendan J. Godley

**Affiliations:** Centre for Ecology and Conservation, University of Exeter, Penryn, Cornwall, United Kingdom; Monash University, Australia

## Abstract

**Background:**

Conservation of marine ecosystems will require a holistic understanding of fisheries with concurrent spatial patterns of biodiversity.

**Methodology/Principal Findings:**

Using data from the UK Government Vessel Monitoring System (VMS) deployed on UK-registered large fishing vessels we investigate patterns of fisheries activity on annual and seasonal scales. Analysis of VMS data shows that regions of the UK European continental shelf (i.e. Western Channel and Celtic Sea, Northern North Sea and the Goban Spur) receive consistently greater fisheries pressure than the rest of the UK continental shelf fishing zone.

**Conclusions/Significance:**

VMS provides a unique and independent method from which to derive patterns of spatially and temporally explicit fisheries activity. Such information may feed into ecosystem management plans seeking to achieve sustainable fisheries while minimising putative risk to non-target species (e.g. cetaceans, seabirds and elasmobranchs) and habitats of conservation concern. With multilateral collaboration VMS technologies may offer an important solution to quantifying and managing ecosystem disturbance, particularly on the high-seas.

## Introduction

For global commercial fisheries to maintain a sustainable future [1], [2] there is a need to develop and implement ecosystem management plans that enable managed exploitation of fish stocks while mitigating against bycatch [3]–[7]. These goals are most likely to be achieved through the development of spatially explicit models on the distribution of fisheries activity, commercially desirable fish stocks and non-target species and habitats.

Knowledge regarding the spatial ecology of non-target species of conservation concern (e.g. cetaceans, elasmobranchs, turtles and seabirds) is ever-growing from boat and aerial surveys [8], an increasing array of electronic tagging and tracking methods [9], [10], plus molecular and other forensic techniques [11], [12]. Analyses of capture records from vessels carrying independent observers have both elucidated the ecology of non-target species but also provided effort-corrected and temporally and spatially relevant insights into the magnitude of impacts of different gear types [13]–[15].

Creating a generalised, yet spatially and temporally explicit, understanding of fisheries effort with which to evaluate potential capture of target stocks and minimise putative risk to non-target species and habitats is far from trivial. Information on the at-sea distribution and behaviour of fishing vessels may be obtained from routine and opportunistic surveillance by enforcement agencies using boats and planes, but these approaches lack spatial and/or temporal coverage. Catch-book data can be used but are subject to potential biases in reporting [5]. Vessel Monitoring Systems (VMS) deployed by several nations on large commercial fishing vessels [16] could however provide patterns of fisheries activity as they have good temporal and spatial coverage and are catch-book and vessel-master independent.

In the Europe Union, VMS operates on larger vessels of Member States fishing fleets (≥15 m overall length). Such vessels employ a range of fishing techniques to exploit demersal and pelagic fish species (e.g. dredging, beam trawling, pair-trawling, gill netting and longlining). These techniques have their respective degrees of selectivity for both their intended catch species but also non-target species and variable impacts on habitats. For example, small cetacean bycatch is commonly associated with bottom set gill-netting and pair trawling [17], whereas dredging is more harmful to benthic habitats [18].

Here we investigate the utility of data from the UK VMS to describe patterns of at-sea space use by large UK-registered fishing vessels. Such data may ultimately inform seascape scale conservation by feeding into marine spatial planning activities [19] that should ensure sustainable persistence of commercial fisheries and effective mitigation of putative risk to species and habitats of conservation concern.

## Results

**Figure 1:**
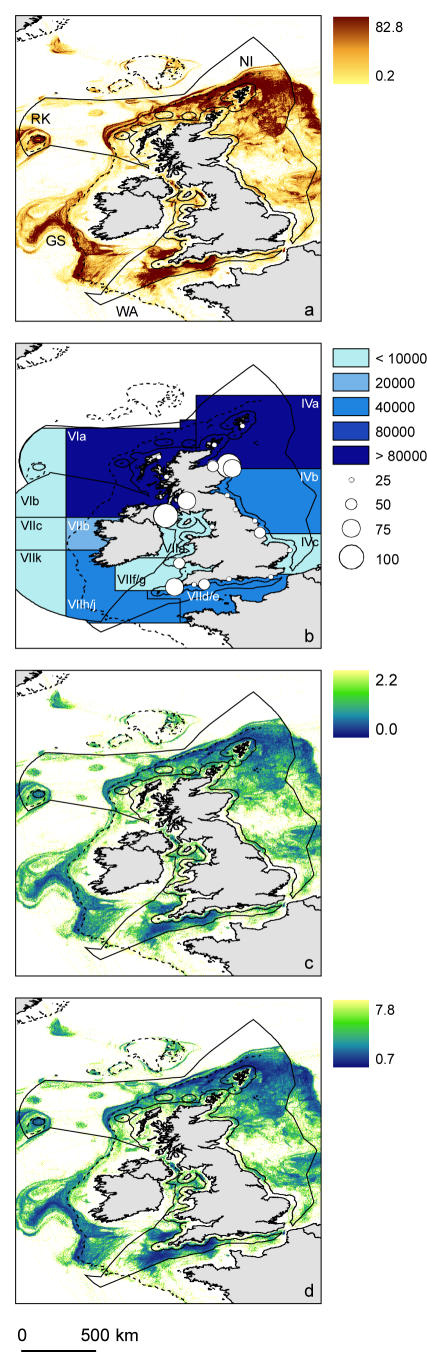
a) Mean annual spatial distribution of fisheries activity derived from VMS records using a simple speed filter. The colour scale indicates the mean annual number of VMS derived data points within 9 km^2^ pixels, solid line circumscribes the UK declared fishing zone, broken line is 200 m depth contour. Regional labels: Western Channel (WA), Goban Spur (GS), Rockall (RK) and Northern North Sea (NI). b) Tonnes of fish (demersal and pelagic) landed by UK registered vessels from the shown ICES statistical reporting boxes. Total number of vessels registered at main UK fishing ports greater than 17 metres in overall length (filled circles). All vessels for Northern Ireland have been mapped to Belfast. c) Coefficient of variation of the mean annual distribution of fisheries activity, lighter colours indicate areas of greatest variability in space-use, darker areas indicate regions of consistent space-use on annual time-scales. d) Coefficient of variation of the mean monthly distribution of fisheries activity, lighter colours indicate areas of greatest variability in space-use, darker areas indicate regions of consistent space-use on monthly time-scales.

Mapping of VMS data highlights considerable heterogeneity in space use (Figure 1a). Regions of the UK continental shelf and the European continental shelf-edge (i.e. Western Channel and Celtic Sea, Northern North Sea and the Goban Spur) receive appreciable fisheries pressure. Shelf habitats (≥25 m and ≤150 m depth, 85% of the UK declared fishing zone), received 64.1% of fisheries activity. Shelf-edge habitats (≥150 m and <250 m depth), which are not exclusively within the UK declared fishing zone, received 16.6% of fisheries activity.

To validate the presented fishing patterns (Figure 1a) we mapped sea fisheries statistics for landings of demersal and pelagic fish (Figure 1b), by area of capture, landed by UK-registered vessels during 2004 (presented in ICES statistical reporting boxes) [20]. When comparing these figures to the mean annual pattern of fisheries activity (Figure 1a) we see there is a statistically significant correlation (Spearman rank order correlation *r_s_* = 0.6, P<0.05) between the levels of fishing activity and declared fish landed. Also insightful is the general correlation of fisheries hotspots with the magnitude of the number of vessels registered in proximate harbours (represented by the filled circles); for example, Newlyn in the southwest and Peterhead and Northern Ireland in the northeast and northwest respectively (Figure 1b).

It is highly likely that VMS data plots fishing activity with a much greater degree of precision than inferences that could be made from catch-book data. Is this high resolution picture predictable across years and across seasons as would be needed for efficient design of spatially explicit management? When we spatially map coefficient of variation (CV) among years (Figure 1c, annual mean maps in Figure S3) and across months (Figure 1d, mean monthly maps in Figure S4) it is clear that hotspots of fisheries activity are consistent through time.

## Discussion

VMS was initially conceived to assist in the monitoring and control of fisheries activities and was legislated prior to changes in EU common fisheries policy [21], which emphasised a greater focus on understanding the effects of fishing at an ecosystem level. We show that VMS, while not designed to understand putative risk to marine ecosystems, can aid EU Member State's obligations under the Common Fisheries Policy and Habitats Directive to manage ecosystem impacts of fisheries. VMS mapping generates a spatially and temporal explicit view of fisheries activity at a far greater resolution than catch-book statistics. VMS data have great potential to highlight areas where the success of ecosystem management plans may be investigated.

The importance of the identified centres of fisheries activity (i.e. Western Channel and Celtic Sea, Northern North Sea and the Goban Spur) can be explained from biological and physical oceanographic perspectives. These are regions where seafloor topography and currents set up physical features that act to support upwelling, enhanced mixing, input of nutrient rich waters, or aid the development and maintenance of frontal systems that aggregate biological matter [22], [23]. These features support primary and secondary productivity, the resulting energy of which is transferred to higher trophic levels within regional food webs. Such factors highlight why fisheries and many marine megavertebrate species seeking prey occupy similar habitats.

With the increased resolution of spatio-temporal patterns of fisheries a step improvement in knowledge of the spatial distribution of species and habitats of conservation concern is required. This requirement has been met, in part, by UK and EU funded research on small cetaceans [8], [24] and seabirds [25]. There are however statistical problems preventing the data from such studies being used as a full correlative data layer to compare with patterns of fisheries activity (i.e. merging species specific distribution and abundance data produced from differing survey methodologies; pers. comm. Simon Northridge–NERC Sea Mammal Research Unit, UK). More recently SCANS II, funded through the EU-LIFE program and participating EU Member States, has aided a more quantitative understanding of the spatial distribution and abundance of cetaceans [26]. Seasonal patterns of distribution and abundance are however still lacking and given the seasonal nature of fisheries such information is required to gain a coherent understanding of putative risk.

Although the VMS approach is a step forward in aiding the development of ecosystem management plans, there are a number of important caveats that must be considered in the interpretation of our findings, which suggest future directions for research. The fisheries activity maps are indicative of the spatial and temporal distribution of large UK-registered fishing vessels only. The patterns are therefore biased towards more offshore fishing activity and represent only a subset of the UK fleet. In addition, we only present data from the UK-registered fleet and not from other EU Member States operating in UK waters. The lack of these data does not detract from the utility of VMS data in providing a spatially and temporally explicit understanding of fisheries activity. Their absence does, however, highlight the need for integration with VMS data from other Member State vessels operating in UK domestic waters. A synoptic European view of fisheries activity will be essential for understanding the relationship between fisheries and migratory target and non-target species as they move seasonally between the waters of distant Member State.

The absence of metadata in the UK VMS on vessel gear type required us to use assumptions on movement speeds that most likely characterise fishing behaviour across several fishing methods employed by larger fishing vessels. In using a narrow range of speeds we believe we have been parsimonious in our estimation of when a vessel might be engaged in fishing. The common factor that a fishing vessel travels at slower speeds during fishing, gear deployment and retrieval, be it demersal or pelagic gear, provides a characteristic, albeit coarse, signal upon which to partition data. Expanding and contracting the width of the speed filter has the effect of widening or constricting the observed spatial patterns; what remain consistent are the identified centres of fisheries activity. Identification of these areas, their spatial range and their seasonality, provides important information for spatial management plans that could seek to manage fish stock extraction while mitigating risk to non-target species and habitats.

Not all fisheries techniques pose the same degree of risk to species and habitats of conservation concern, yet this lack of metadata does not prevent a coarse spatial interpretation of the putative risk posed to these groups as gear types, with their associated risks, are commonly deployed in known depths of water over particular habitat types. Moreover, non-target species adopt fairly predictable habitat utilisation patterns and physical habitats that represent areas of increased biodiversity can be mapped [27]. In deeper off-shore waters, such as those of the continental shelf-break, fishing vessel activity most likely represents pelagic techniques such as mid-water trawling and purse-seining. In shallower waters, fisheries activity will increasingly involve demersal techniques including bottom trawls and dredging. In the absence of robust metadata it may however, be possible to use behavioural rules on turning angles, bathymetry in the area of operation and information on movement patterns to help assist in more accurately characterising and spatially placing fishing behaviour. The development and implementation of electronic logbook system for fisheries [28], [29] may make a substantial contribution in European waters; providing spatially explicit information on gear deployment, duration of fishing and capture of target and non-target species.

Recent work to describe trawl intensity received by the seabed [30], [31] highlights additional uses of VMS for ecosystem management. Such approaches help describe the amount of disturbance an area receives. When integrated with knowledge of benthic habitat type [27] and derived habitat sensitivity, VMS data might provide better ways to manage the seabed and the fish stocks they support. VMS may also have utility in assisting the designation and subsequent measurement of the effectiveness of Marine Protected Areas that function to conserve both target stock spawning biomass and non-target species and habitats. VMS could assist in optimally selecting such areas.

Notwithstanding the caveats, the simple and coherent patterns of habitat occupation by fishing vessels presented here suggest that fishing activity could be managed on a more finely resolved spatial and temporal basis. Furthermore, with multilateral collaboration VMS technologies may offer an important solution to quantifying and managing ecosystem disturbance particularly on the high-seas, which has become evermore important as fisheries move into deeper [32] and more distant waters.

## Materials and Methods

### Vessel Monitoring System

The Vessel Monitoring System (VMS) is an automated method of recording the location of fishing vessels at sea. The system consists of a tamper-proof installation onboard fishing vessels registered in the UK and was introduced under European Commission legislation (EC 686/97). Each unit consists of a global positioning satellite (GPS) receiver; a satellite transmitter and a power backup that will last approximately 72 hours [33]. From the year 2000, these units were mandatory for fishing vessels greater than 24 metres overall length, from 2004 they were mandatory for vessels greater than 18 metres length and from 2005 for vessels greater than 15 metres overall length. VMS units are required to report 99% of all locations accurate to within 500 metres [33], [34]. VMS units operating in UK waters report location and ancillary data (i.e. speed and heading), via satellite communication, on a 2-hour duty cycle to the UK Fisheries Monitoring Centre (FMC). The FMC may request the location of a fishing vessel at any time from the VMS unit. VMS units can also be tasked to increase the reporting frequency within certain regions or within the waters of other EU Member States.

### VMS dataset

VMS data were obtained from the UK Sea Fisheries Inspectorate in 2005 (now the Marine and Fisheries Agency of the Department for Environment, Food and Rural Affairs). This dataset contained 5,788,188 records. Each record contained geographic coordinates in decimal degrees (World Geodetic System 1984 format) an accompanying time stamp in UTC and a vessel identification number. All received data were anonymous with respect to their vessel registration numbers, dimensions and administrative ports. The mean number of VMS records per year (see Figure S1a) was 840,182±60,346 SD (range 756,863 to 926,363). Filters were applied to the VMS dataset to remove: a) erroneous geographic records outside the range 90°S to 90°N, −180°W to 180°E, b) records outside the 5 year study period, set to be 01-01-2000 to 31-12-2004, and c) records with elevations greater than 50 metres above sea-level as determined from the TerrainBase digital elevation model [35]. The number of vessel identification numbers appearing in the dataset declined annually (from 422 in 2000 to 334 in 2004, see Figure S1b); however, new identification numbers were introduced annually to the dataset during the study period (2001 *n* = 23, 2002 *n* = 26, 2003 *n* = 20 and 2004 *n* = 32).

### Route reconstruction

Fishing trips were reconstructed as follows: a 5 km buffer zone was constructed around the coastline of Europe, this was used to determine when vessels were leaving or nearing ports. All records belonging to a vessel were assigned a logical flag (1 or 0) to indicate whether they were inside or outside this coastal buffer zone. The start and finish of a fishing trip was determined when a vessel moved out of and back into the zone with respect to time. Records occurring within the buffer zone were discarded. A speed filter was applied to remove improbable locations; this process removed locations necessitating travel speeds greater than 100 km hr^−1 ^(∼55 knots) between time adjacent locations. The filter was triggered on 1,015 trips and removed 6,891 records.

Potential trips were discarded if they contained ≤3 VMS records, or were ≤6 hours in duration or had transmission breaks ≥5 days; removing 28,800; 12,121 and 168,549 records respectively (in total 3.6% of the original dataset). It is likely that these filters remove some legitimate fishing trips of short duration and may underestimate near-shore fishing effort. However, they were required to minimising the degree of visual supervision needed to manage this large dataset while maximising retention of VMS data. Post filtering the dataset contained 56,434 fishing trips (see Figure S2).

The modal frequency of record transmission was 2 hours (see Figure S1c). To ensure temporal consistency among data, all trips were re-sampled where necessary to a 2 hour±15 minutes frequency using great circle, speed-appropriate, principles. This process maximised the retention of transmitted records, only filling temporal gaps where necessary and resulted in a 14% reduction from pre-treated data, making available 3,635,855 data points. The mean net change in the number of data points following this temporal alignment process for each trip was −8.9; 28,320 trips experienced a net addition, receiving an average of 10±19 data points, 13,986 trips experienced a net reduction, losing an average of 56±198 VMS records; 14,776 trips experienced no adjustment in their temporal frequency.

### Vessel behaviour

A speed rule was used to distinguish fishing from steaming or near-stationery movement. It was necessary to construct derived speeds for all VMS records as prior to 1-1-2006 transmission of speed and heading was not mandatory [34]. Derived speeds represent the speed of movement between time adjacent records within a fishing trip. We compared transmitted vessel speeds available from 40,681 fishing trips (3,126,213 VMS records) to comparative derived speeds to ensure that these speeds were closely mirrored. The process identified 78.9% of fishing trips yielded statistically significant positive correlations between transmitted and derived speeds (Pearson correlation coefficient; p≤0.05; mean r^2^ = 0.6 for all fishing trips). The speed filtering process assigned 1,710,725 data points (47% of available data) as representing fishing activity (see Figure S2).

The upper and lower speed thresholds for determining fisheries activity were influenced by the frequency distribution of vessel speeds (see Figure S1d), and from published values [30], [31], [36]. As the UK VMS database retains incomplete data on vessel gear type and vessels can change their gear seasonally it was necessary for the speed rule to encompass many types of fisheries activities, for example beam trawling, gill netting and longlining. The lack of metadata prevents VMS data from being partitioned by gear type. Fishing activity was therefore assigned to all vessels travelling at speeds ≥3.0 and ≤10.0 km h^−1^, (∼1.5 to 5.5 knots). While this approach is a coarse manner in which to filter the data, the assigned limits circumscribes the speeds at which larger vessels move while undertaking fisheries activities.

### Mapping fisheries activity

Fisheries activity was gridded at a spatial resolution of 9 km^2 ^(3 km by 3 km pixel) by summing the number of VMS derived data points coincident to each pixel over monthly and annual scales.

## Supporting Information

Figure S1 a) Number of VMS records (x104) per year, b) number of vessel identification numbers active each year (filled bars) and cumulative increase in vessel identification numbers appearing each year in the VMS dataset (empty bars), c) frequency histogram of time elapsed (hours) between transmission of time adjacent records for all vessels in the 5 year VMS dataset, d) frequency histogram of transmitted and derived speeds (filled and empty bars respectively) for 3,126,042 VMS records, and e) frequency histogram of transmitted and derived headings (filled and empty bars respectively) for 3,126,042 VMS derived data points.
(0.03 MB DOC)

Figure S2 Data handling/filtering process applied to the VMS dataset.
(0.05 MB DOC)

Figure S3 Mean annual maps of fishing activity (vessels moving ≥3 and ≤10 km h 1) for the period 2000–2004. Maps show the mean number of data points at each pixel, where darker colour indicates greater number of visits by vessels travelling at speeds most likely to indicate fisheries activity.
(4.12 MB EPS)

Figure S4 Mean monthly maps of fishing activity (vessels moving ≥3 and ≤10 km h 1) for the period 2000–2004. Maps show the mean number of data points at each pixel, where darker colour indicates greater number of visits by vessels travelling at speeds most likely to indicate fisheries activity.
(9.28 MB EPS)
