# Supplementary material for: A Step Towards Seascape Scale Conservation: Using Vessel Monitoring Systems (VMS) to Map Fishing Activity
Source: PLoS One. 2007 Oct 31;2(10):e1111. doi: 10.1371/journal.pone.0001111 (PMC2040201; doi:10.1371/journal.pone.0001111)
Supplement: Figure S1 — a) Number of VMS records (x104) per year, b) number of vessel identification numbers active each year (filled bars) and cumulative increase in vessel identification numbers appearing each year in the VMS dataset (empty bars), c) frequency histogram of time elapsed (hours) between transmission of time adjacent records for all vessels in the 5 year VMS dataset, d) frequency histogram of transmitted and derived speeds (filled and empty bars respectively) for 3,126,042 VMS records, and e) frequency histogram of transmitted and derived headings (filled and empty bars respectively) for 3,126,042 VMS derived data points. (0.03 MB DOC) [file pone.0001111.s001.doc]

d

a

b

c

e
