# Supplementary material for: A Step Towards Seascape Scale Conservation: Using Vessel Monitoring Systems (VMS) to Map Fishing Activity
Source: PLoS One. 2007 Oct 31;2(10):e1111. doi: 10.1371/journal.pone.0001111 (PMC2040201; doi:10.1371/journal.pone.0001111)
Supplement: Figure S2 — Data handling/filtering process applied to the VMS dataset. (0.05 MB DOC) [file pone.0001111.s002.doc]

| Description / procedure | Change | Remaining data | Vessels/ trips |
| --- | --- | --- | --- |
| Dataset received from UK Sea Fisheries Inspectorate | NA | 5,788,188 | 691 vessels |
| Records with anomalous geographic positions removed | -257 | 5,787,931 | 691 vessels |
| Records outside study period removed | -184,675 | 5,603,256 | 626 vessels |
| Records outside study region removed | -288,500 | 5,314.756 | 618 vessels |
| Records with inappropriate elevations, > 50 m, removed | -86,260 | 5,228,496 | 616 vessels |
| Records with erroneous headings, > 360°, removed | -186 | 5,228,310 | 616 vessels |
| Vessels contributing 10 or less records removed | -97 | 5,228,213 | 542 vessels |
| Time duplicated records removed | -28,678 | 5,199,535 | 542 vessels |
| Conversion of each vessel’s data to independent trips | | | |
| Removal of records within buffer zone | -767,381 | 4,432,154 |  |
| Removal of records with improbable derived speeds | -6,891 | 4,425,263 |  |
| Removal of potential trips with ≤ 3 VMS records | -28,800 | 4,384,342 |  |
| Removal of potential trips ≤ 6 hours in duration | -12,121 | 4,394,528 |  |
| Removal of potential trips with transmission breaks > 5 days | -168,549 | 4,225,979 |  |
| Trips passing rule based filtering |  | 4,225,979 | 56,434 trips |
| Temporal alignment of all fishing trips to 2 hour ± 15 minute frequency | | | |
| N data points with speeds < 3 km h^-1^ |  | 1,256,565 |  |
| N data points with speeds ≥ 3 & ≤ 10 km h^-1^ (fishing) |  | 1,710,725 |  |
| N data points with speeds > 10 km h^-1^ (steaming) |  | 668,565 |  |
